# Increase of intestinal bacterial sialidase activity exacerbates acute colitis in mice

**DOI:** 10.3389/fmolb.2022.1075459

**Published:** 2022-12-09

**Authors:** Tobias Hasler, Leticia Tavares-Gomes, Sereina Gut, Meghna Swayambhu, Mario Gysi, Martin Hausmann, Natasha Arora, Thierry Hennet

**Affiliations:** ^1^ Institute of Physiology, University of Zurich, Zurich, Switzerland; ^2^ Institute of Forensic Medicine, University of Zurich, Zurich, Switzerland; ^3^ Department of Gastroenterology and Hepatology, University Hospital Zurich, University of Zurich, Zurich, Switzerland

**Keywords:** glycosidase, glycoside hydrolase, sialic acid, siglec, dextran sulfate sodium, gut microbiota

## Abstract

The availability of endogenous and dietary carbohydrates in the gastrointestinal tract influences the composition of the gut microbiota. Carbohydrate foraging requires the action of bacterially-encoded glycoside hydrolases, which release mono- and oligosaccharides taken up as carbon sources by multiple microbial taxa. In addition to providing nutrients to the microbiota, the cleavage of host glycans by bacterial glycoside hydrolases may alter the properties of surface glycoproteins involved in cell adhesion and activation processes in the gut lumen. To investigate the impact of bacterial glycoside hydrolase activities on the gut microbial composition and on host glycans during colon inflammation, we increased local glycoside hydrolase activity by supplementing mice with recombinant *E. coli* expressing specific sialidase, fucosidase and rhamnosidase enzymes during acute colitis induced by dextran sulfate sodium ingestion. Whereas increased fucosidase and rhamnosidase activity did not alter the course of colitis, increased sialidase activity exacerbated disease severity. The effect of increased sialidase activity on inflammation was not caused by changes in the microbial composition given that a similar shift in gut bacteria occurred in all groups of mice supplemented with recombinant *E. coli.* Increased sialidase activity in the colon of treated mice however significantly altered the distribution of sialic acid on mucosal glycans. Treatment of lamina propria dendritic cells with bacterial sialidase also strongly decreased the density of sialylated ligands to anti-inflammatory siglec lectins, indicating that the remodeling of surface sialylation caused by increased sialidase activity likely accounts for the observed exacerbation of acute colitis in mice.

## Introduction

The gastrointestinal tract is home to a diverse community of microbes, numbering in the trillions (Matijasic et al., 2020). The gut microbiota mainly consists of the phyla Bacteroidetes and Firmicutes, while members of Actinobacteria, Verrucomicrobia, Fusobacteria and Proteobacteria represent a minor fraction (Shreiner et al., 2015; Rinninella et al., 2019). The gut microbiota plays a vital role in human health, supplying essential nutrients, training the immune system, and helping to protect against pathogens. The gut microbiota is also involved in the host metabolism and has been linked to a variety of chronic diseases (Hou et al., 2022). As a complex ecosystem, the gut microbiota is in constant competition for nutrients. This competition between gut microbes can lead to durable shifts in the composition of the microbiota and hence to various negative health outcomes for the host (Coyte and Rakoff-Nahoum, 2019; Schroeder, 2019). For example, dysbiosis caused by imbalance of the gut microbiota is linked to numerous diseases, including obesity, inflammatory bowel disease, and cancer (Gomes et al., 2018; Gomaa, 2020). Therefore, understanding the competition for nutrients within the gut microbiota is essential for promoting gut health and preventing disease (Sonnenburg et al., 2005).

To acquire and process nutrients, gut microbes express a multitude of genes, including a vast group of carbohydrate-active enzymes, which are required to digest complex oligo- and polysaccharides. In addition to digesting dietary carbohydrates, these enzymes are also active on the thick mucus layer lining intestinal surfaces, which mainly features the gel-forming mucins MUC2, MUC5AC, and MUC5B (Desai et al., 2016; Melhem et al., 2021). Bacterial carbohydrate-active enzymes catalyze the release of monosaccharides from dietary carbohydrates and mucus glycans into the gut lumen. The carbohydrate-active enzymes involved in the digestion of the intestinal mucus belong mainly to glycoside hydrolases (GHs) of type sialidase, fucosidases, galactosidase and hexosaminidase (Belzer, 2022). The majority of GHs present in the gut are produced by members of the phyla Firmicutes and Bacteroidetes (El Kaoutari et al., 2013). Monosaccharides released into the gut lumen also promote the growth of bacterial taxa lacking dedicated GHs for the breakdown of carbohydrates, thus leading to cross-feeding pathways (Berkhout et al., 2022). The beneficiaries of monosaccharide cross-feeding are often pro-inflammatory, pathogenic bacteria such as *Escherichia coli* or *Salmonella thyphimurium* (Ng et al., 2013; Huang et al., 2015).

The cleavage of host glycans by bacterial GHs may also alter carbohydrate structures involved in cell adhesion, trafficking and activation. Especially fucosylated and sialylated epitopes are recognized by endogenous lectins, such as selectins and sialic acid-binding immunoglobulin-like lectins (siglec), which regulate leukocyte trafficking and activation, respectively (Crocker et al., 2007; McEver, 2015; Tvaroska et al., 2020; Murugesan et al., 2021). The removal of fucose by bacterial fucosidases may influence the signaling of the immunomodulatory C-type lectin DC-SIGN on dendritic cells (DCs) that modulate the pro-inflammatory responses of DCs (van Gisbergen et al., 2005; Garcia-Vallejo et al., 2014). Similarly, the action of bacterial sialidases in the gut lumen may decrease the density of siglec ligands, thereby lowering the anti-inflammatory activity of siglecs (Chen et al., 2011; Chang et al., 2012). Sialidase activity has been shown to increase the activation of immune cells in the context of siglec-mediated signaling in cancer immunotherapies (Gray et al., 2020; Läubli et al., 2021). Accordingly, it is possible that increased sialidase activity decreases the activation-threshold of leukocytes, thereby increasing inflammation in the gut, as suggested by our previous results (Huang et al., 2015).

To investigate the impact of increased bacterial fucosidase and sialidase activity on intestinal physiology and inflammation, we colonized mice with *E. coli* over-expressing selected bacterial GHs. Using this supplementation approach, we were able to compare the effects of increased GH activity on the development of acute colitis induced by dextran sulfate sodium (DSS) ingestion, thus demonstrating the pro-inflammatory action of sialidase activity.

## Results

**FIGURE 1:**
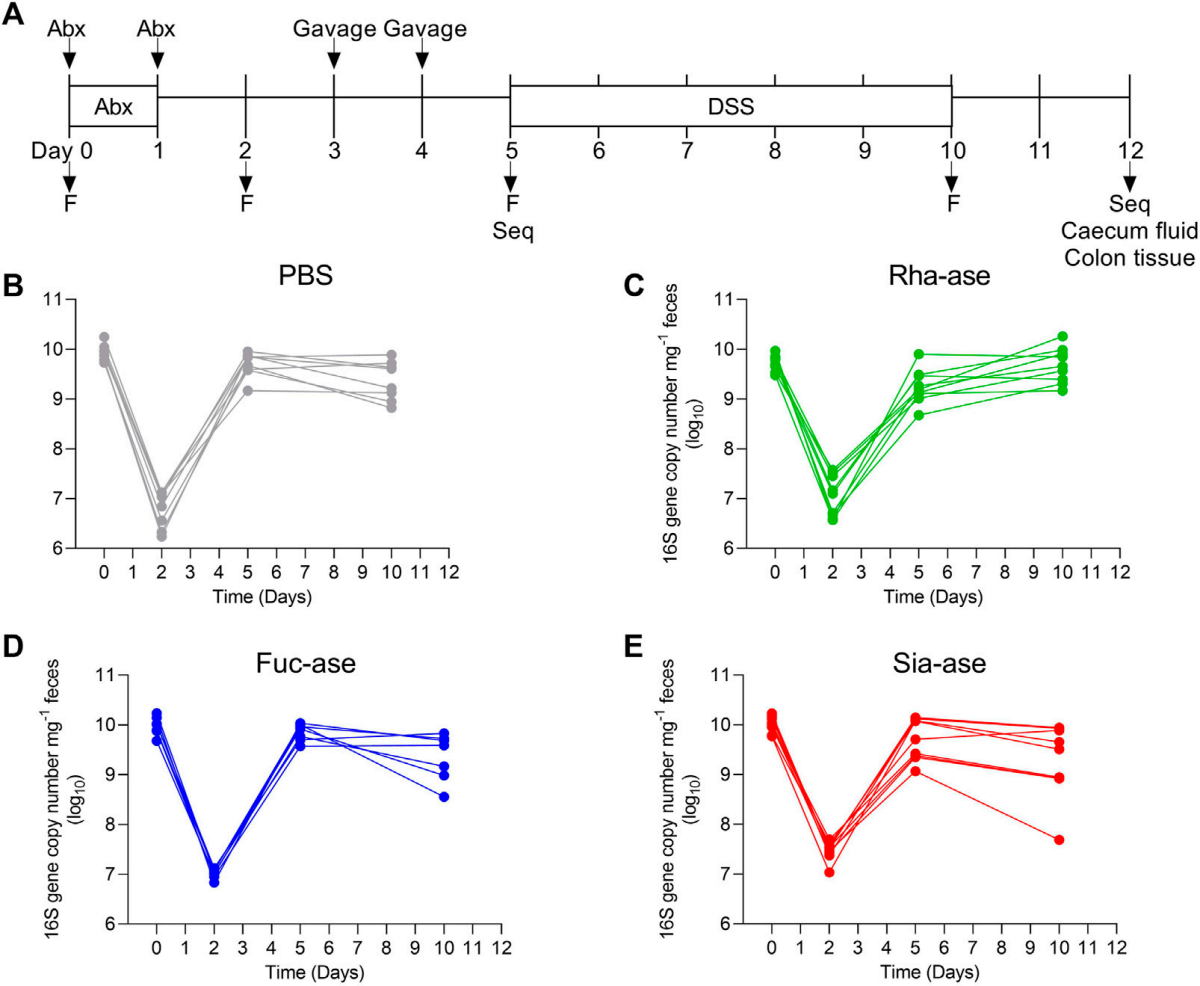
Supplementation of mice with recombinant *E. coli*. **(A)** Timetable of mice supplementation. F, collection of fecal pellets; Seq, 16S rRNA sequencing of bacterial DNA. Total bacteria abundance of **(B)** PBS-, **(C)** Rha-ase-, **(D)** Fuc-ase-, and **(E)** Sia-ase-supplemented mice.

To assess the effects of increased bacterial sialidase and fucosidase activity in the gut, we cloned and expressed a series of candidate genes from various *Akkermansia*, *Bacteroides* and *Ruminococcus* species represented in the carbohydrate-active enzyme (CAZy) families GH29 and GH95 for fucosidases, and GH33 for sialidases. For downstream heterologous expression in *E. coli*, we selected enzymes that showed a high specific activity (Table 1, Supplementary Figure S1) and stability across a broad range of temperatures and pH values. For the fucosidase activity, we chose the fucosidase ATCC_03833 encoded by *Ruminococcus gnavus* (UniProt A7B8B7) (Wu et al., 2021). For the sialidase activity, we selected the BVU_4143 sialidase encoded by *Bacteroides vulgatus* (UniProt A6L7T1), which was previously found to expand in the gut of mice during colitis (Huang et al., 2015). We also chose a rhamnosidase enzyme as control GH to compare to the selected fucosidase and sialidase enzymes, given that rhamnose is absent from animal glycans. The BT_1001 rhamnosidase (UniProt Q8A916) from *Bacteroides thetaiotaomicron* has been shown to be active in the cleavage of the pectin rhamnogalacturonan-II (Ndeh et al., 2017). As a bacterial host to express the three GHs, we selected an *E. coli* strain isolated from the gut of an inflamed mouse. This *E. coli* strain, referred to as *E. coli* EHV2, was serotyped as being O7:K-:H7 and was positive for β-glucuronidase and negative for haemolysin and verocytotoxin. The three selected GHs, next described as Rha-ase, Fuc-ase and Sia-ase, were constitutively expressed using vector pSF_OXB20 in *E. coli* EHV2, which resulted in stable rhamnosidase, fucosidase and sialidase activity (Supplementary Figure S1). The resulting recombinant *E. coli* EHV2 were administered by gavage to mice after an initial antibiotic treatment of 2 days to reduce the endogenous gut microbiota and open a niche for the supplemented *E. coli* EHV2. After colonization with recombinant *E. coli* EHV2, mice were treated with 3.5% DSS for 5 days to induce acute colitis (Figure 1A). Antibiotics treatment decreased total bacteria levels by 1000-fold in feces as measured by quantitative 16S rRNA PCR analysis. Bacterial levels returned to normal by day 5 at the beginning of DSS treatment (Figures 1B–E).

**TABLE 1:** Substrate specificity of recombinant bacterial glycoside hydrolases over-expressed in *E. coli*. All substrates were in the pyranos*e* form and linked to 4-nitrophenol (pNP), except NeuNAc, which was linked to 4-methylumbelliferone (4MU). Data represent mean ± s.d. (n = 3).

	Glycoside hydrolase activity [pmol μg^−1^ min^−1^]			
Substrate	Baseline^a^	Rha-ase	Fuc-ase	Sia-ase
α-l-arabinose	401 ± 30	32 ± 3	24 ± 3	26 ± 3
β-d-fucose	88 ± 6	16 ± 1	14 ± 6	17 ± 3
α-d-glucose	55 ± 2	5 ± 1	9 ± 3	26 ± 2
β-d-galactose	372 ± 48	28 ± 2	182 ± 10	53 ± 8
α-l-rhamnose	-^b^	290 ± 8	-	-
α-l-fucose	-	-	7,508 ± 349	-
α-D-NeuNAc	-	-	-	452 ± 41
β-l-arabinose	-	-	-	-
β-l-fucose	-	-	-	-
α-d-mannose	-	-	-	-
β-d-mannose	-	-	-	-
β-d-glucose	-	-	-	-
α-d-galactose	-	-	-	-
α-D-GlcNAc	-	-	-	-
β-D-GlcNAc	-	-	-	-
α-D-GalNAc	-	-	-	-
β-D-GalNAc	-	-	-	-
α-d-xylose	-	-	-	-
β-d-xylose	-	-	-	-

^a^ Baseline activity was measured with His-tag purified proteins from bacteria transformed with empty expression vector.

^b^ -, below detection limit.

**FIGURE 2:**
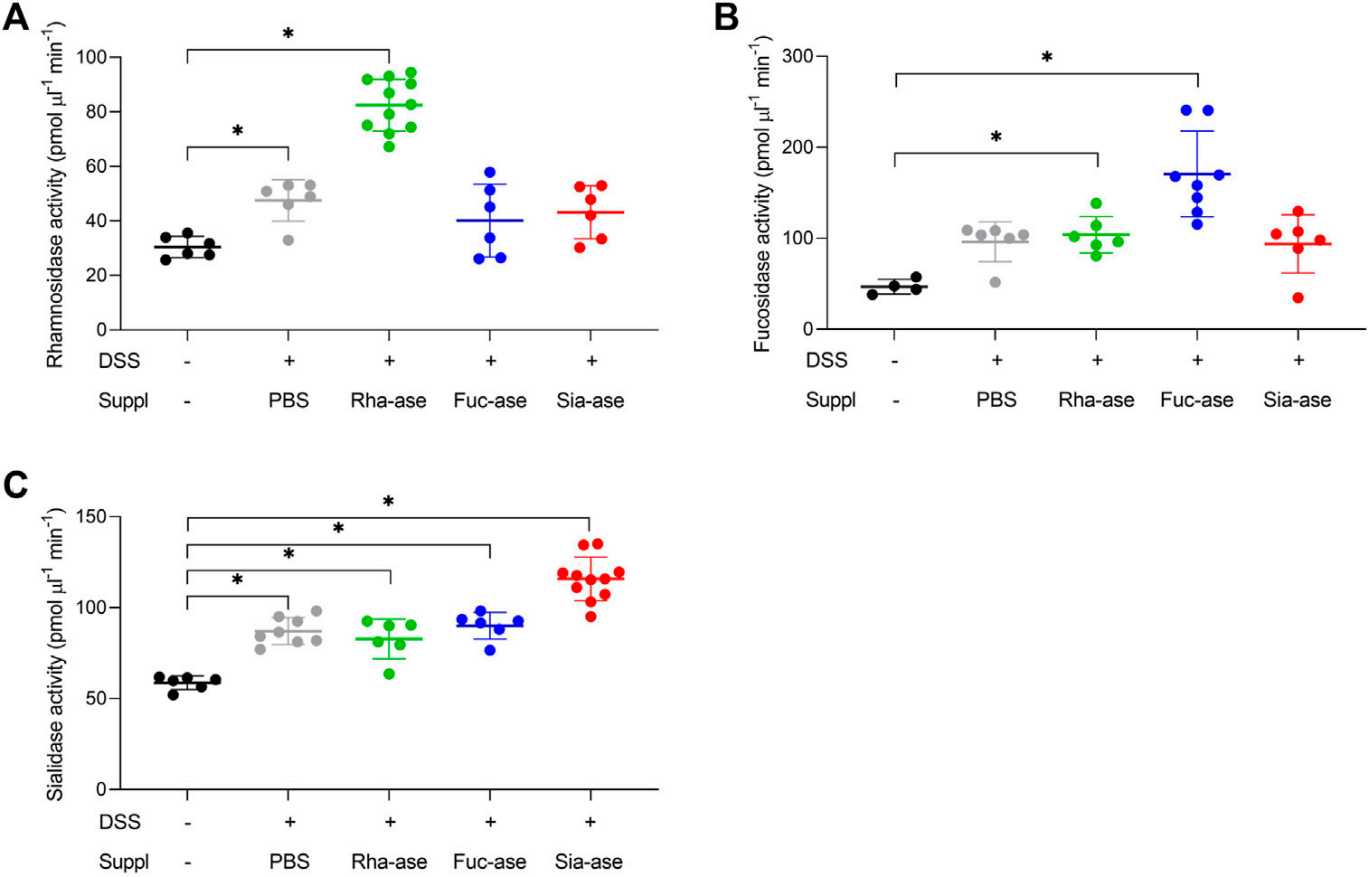
Increased glycoside hydrolase activities in caecum fluid. **(A)** Rhamnosidase activity was determined by colorimetric assay (n = 6–11). **(B)** Fucosidase activity was determined by colorimetric assay (n = 4–8). **(C)** Sialidase activity was determined by fluorogenic assay (n = 6–11). Data represented as mean ± s.d. *p ˂ 0.05 vs. untreated control by one-way ANOVA with Dunnett Post-test.

**FIGURE 3:**
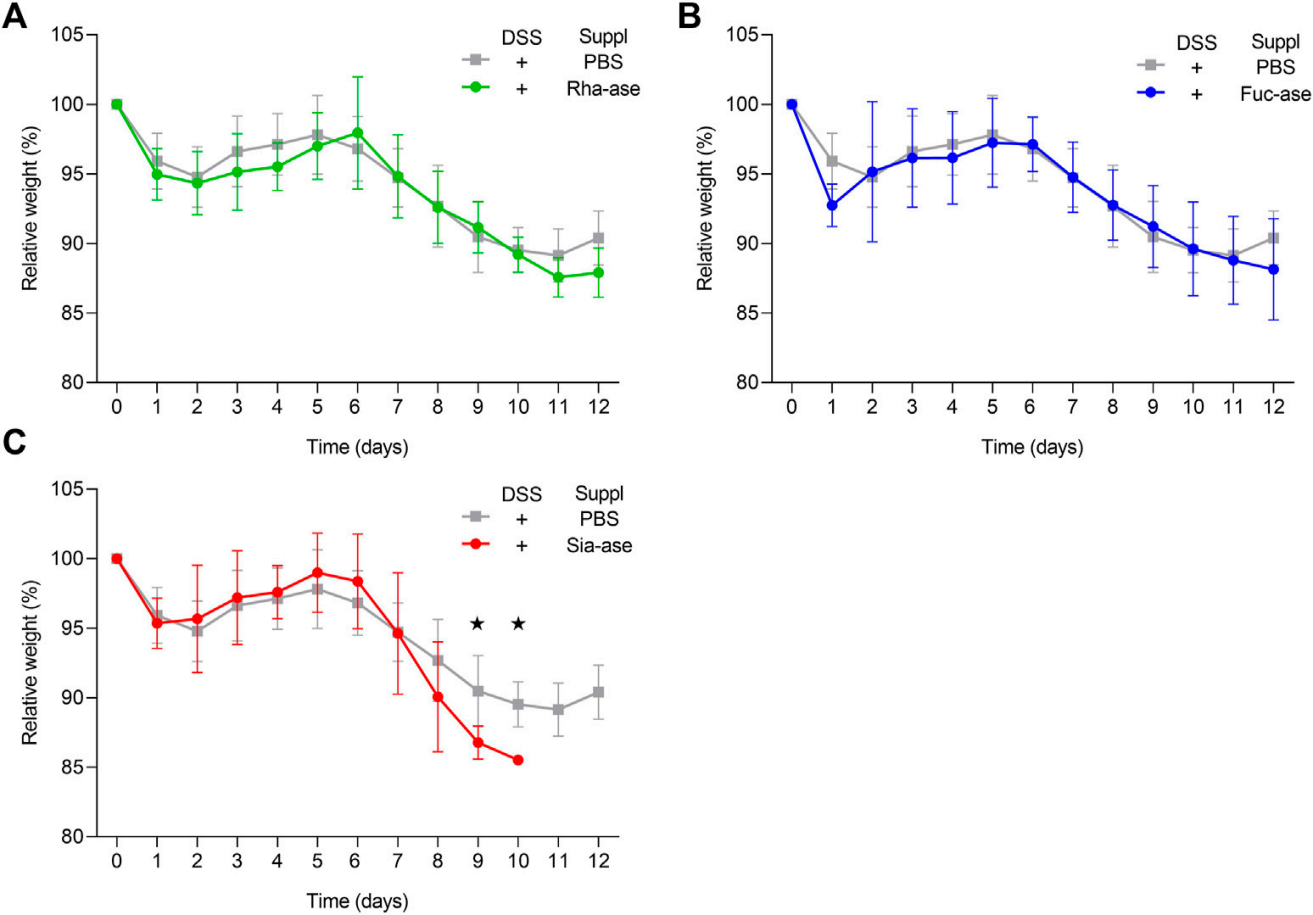
Relative body weight changes in recombinant *E. coli* supplemented mice upon DSS-treatment. **(A)** Relative body weight change of mice supplemented with either Rha-ase expressing *E. coli* (n = 12) or PBS (n = 8). **(B)** Relative body weight change of mice supplemented with either Fuc-ase expressing *E. coli* (n = 8) or PBS (n = 8). **(C)** Relative body weight change of mice supplemented with either Sia-ase expressing *E. coli* (n = 13) or PBS (n = 8). Data are shown as mean ± s.d. *p ˂ 0.05 two-tailed Student’s t-test.

Supplementation of mice with recombinant *E. coli* EHV2 constitutively expressing Rha-ase, Fuc-ase and Sia-ase led to respectively increased rhamnosidase, fucosidase and sialidase activity measured in the caecum fluid until the end of the study by day 12 (Figure 2). Inflammation induced by DSS also increased GH activity, yet to lower levels than achieved by supplementation with recombinant *E. coli* EHV2. The impact of increased rhamnosidase, fucosidase and sialidase activity on DSS-induced colitis was first assessed by monitoring the body weight of treated mice. Antibiotics treatment caused a transitory drop of body weight, which returned to original levels by day 5, when DSS was added to drinking water. Treated mice gradually lost about 10% of weight between day 6 and 11, at which point they started to recover some weight by day 12 (Figure 3). Mice colonized with recombinant *E. coli* EHV2 expressing Rha-ase and Fuc-ase showed the same changes in body weight as the control mice following antibiotics treatment and DSS uptake (Figures 3A,B). By contrast, mice supplemented with *E. coli* EHV2 expressing Sia-ase showed a sharper drop in body weight between days 6 and 10 of the study. This group of mice had to be terminated between day 9 and 10 as they reached the threshold of 85% of their initial body weight (Figure 3C).

**FIGURE 4:**
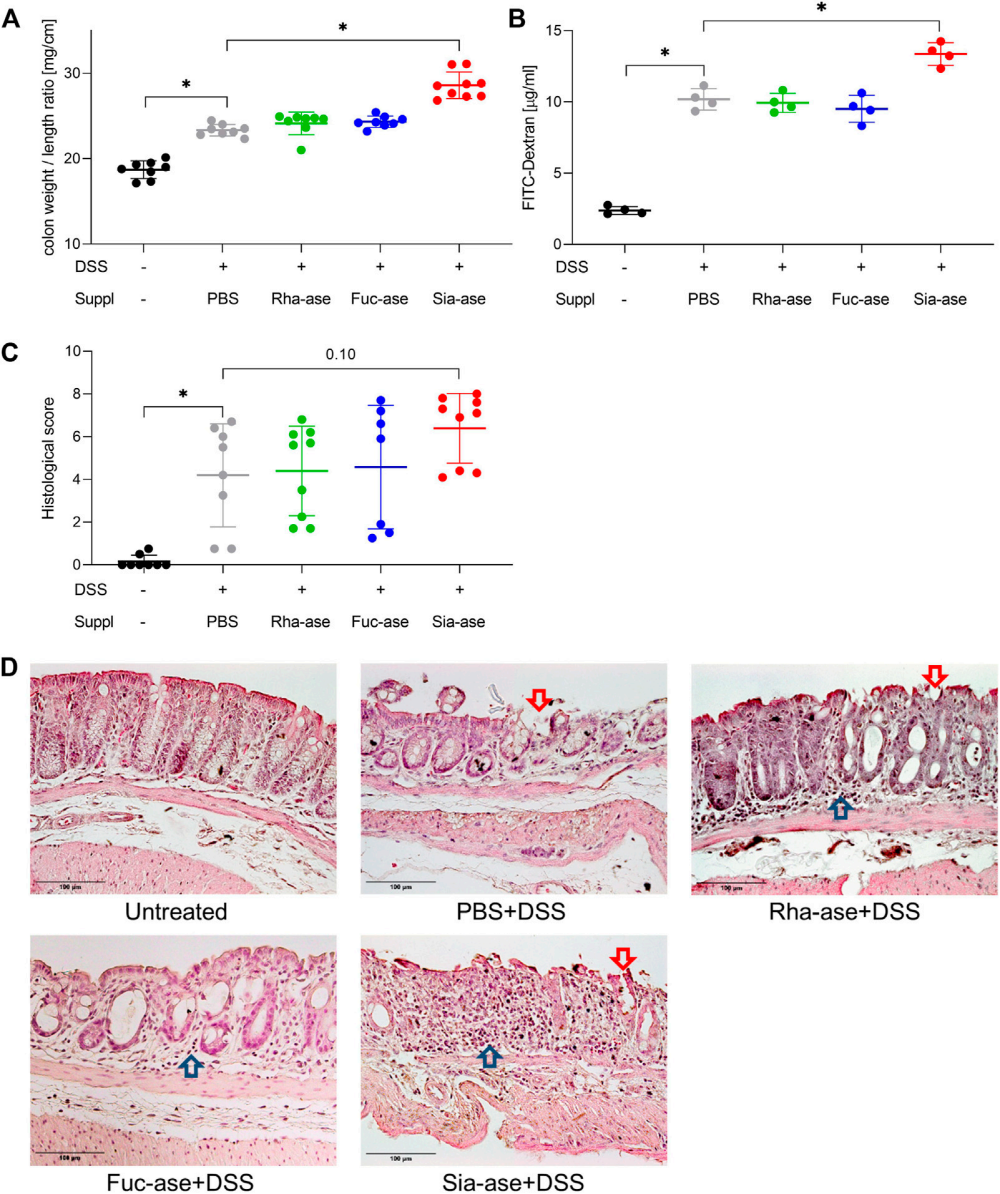
Colon inflammation during DSS-induced colitis in mice with increased GH activity. **(A)** Colon weight to length ratio of supplemented mice. This ratio increases with inflammation (n = 8–9). **(B)** Intestinal permeability determined by serum FITC-dextran levels after gavage (n = 4). **(C)** Histological scoring of inflammation in distal colon tissue (n = 7–9). Data are shown as mean ± s.d. *p ˂ 0.05 vs. DSS treated and PBS supplemented control by one-way ANOVA with Dunnett Post-test. **(D)** Representative images of H&E-stained distal colonic sections isolated from mice at the end of the study, at day 12 or when mice had to be euthanized. Arrows point to altered epithelial barrier (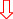) and leukocyte infiltration (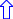). Scale bar 100 µm.

**FIGURE 5:**
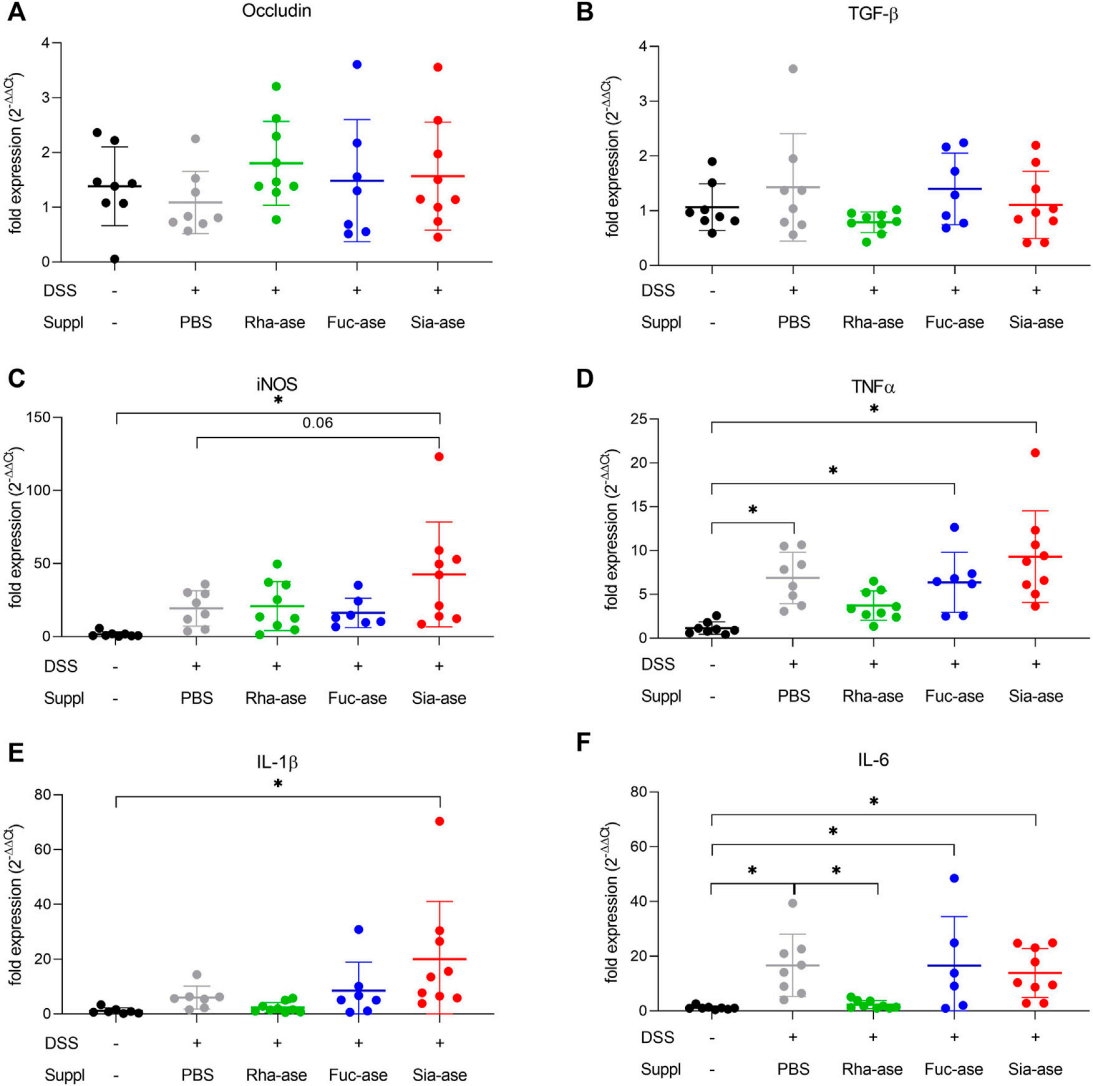
Impact of increased GH activity on inflammation marker expression in distal colon tissue. Expression levels of **(A)** Occludin, **(B)** TGF-beta, and of the inflammation markers **(C)** iNOS, **(D)** TNFalpha, **(E)** IL-1beta, and **(F)** IL-6 were determined by real-time PCR. β-actin was used for normalization and the average of untreated mice was used as reference according to the ∆∆Ct method (n = 7–9). Data are shown as mean ± s.d. *p ˂ 0.05 vs. untreated control by one-way ANOVA with Dunnett Post-test.

In agreement with the observed increased loss of body weight, mice showing increased sialidase activity also had increased colon weight/length ratios (Figure 4A) and increased intestinal permeability as measured by FITC-dextran leakage to the bloodstream (Figure 4B). The groups of mice with increased rhamnosidase and fucosidase activities did not significantly differ from the non-supplemented control group treated with DSS with respect to colon weight/length ratio and intestinal permeability (Figures 4A,B). Histological examination of the inflamed colon from all mouse groups confirmed the typical alterations associated with DSS-induced colitis, such as leukocyte infiltration, loss of epithelial barrier and altered crypts and goblet cell architecture (Figures 4C,D). Scoring of histological alterations confirmed the pathological changes induced by DSS treatment, although without significant differences between the mouse groups despite higher severity scores obtained in mice with increased sialidase activity (Figure 4C). Colon inflammation in DSS-treated mice was also monitored by measuring the expression of marker genes. Transcript levels of occludin, a tight junction protein (Figure 5A), and TGF-β, which is induced in wound healing (Figure 5B), remained unchanged over the course of acute colitis in all mouse groups. Expression of inducible nitric oxide synthase (iNOS) was only significantly increased during DSS-induced colitis in mice with increased sialidase activity (Figure 5C). The pro-inflammatory cytokines TNFα, IL-1β and IL-6 were also mostly overexpressed in inflamed colon tissue of mice with increased sialidase activity, whereas mice with increased rhamnosidase activity showed lower transcript levels for the three cytokines measured (Figures 5D–F), suggesting an effect of rhamnosidase activity on inflammation possibly mediated through changes in microbial composition.

**FIGURE 6:**
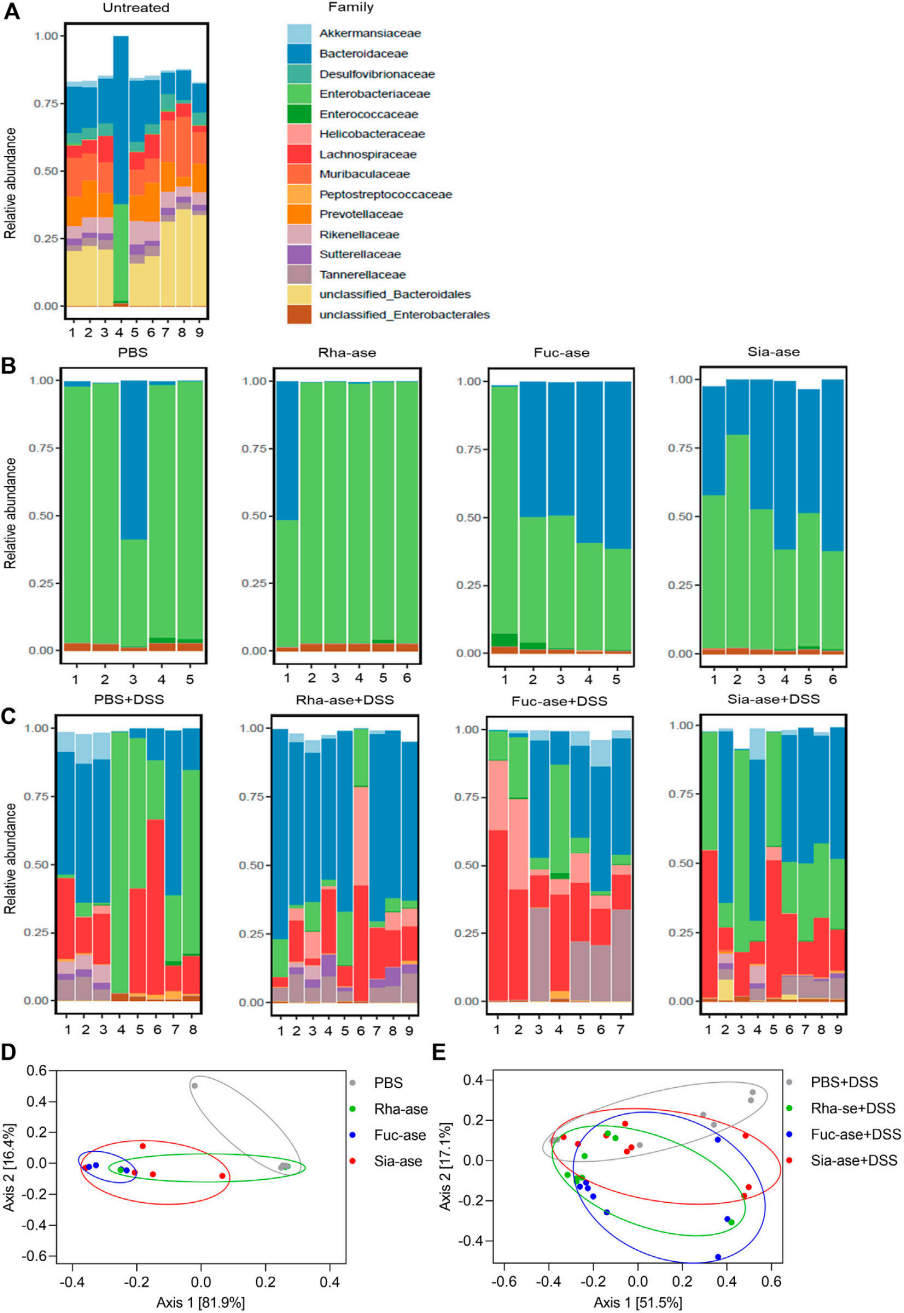
Composition of the gut microbiota in mice supplemented with recombinant *E. coli*. Gut microbiota composition at the family level of **(A)** untreated mice on day 0 (n = 9), **(B)** mice treated with antibiotics and *E. coli* supplementation but without DSS treatment on day 5 of experiment (n = 5–6), **(C)** mice treated with antibiotics and *E. coli* supplementation and undergoing DSS treatment on day 12 or when mice had to be euthanized (n = 7–9). **(D)** Principal coordinate analysis of the gut microbiota composition before DSS treatment on day 5 of experiment (n = 5–6) and **(E)** after DSS treatment on day 12 or when mice had to be euthanized (n = 7–9).

Accordingly, to assess whether the supplementation of mice with recombinant *E. coli* EHV2 and the resulting increased GH activity affected the composition of the gut microbiota, we analyzed the bacterial communities present in treated mice just before DSS administration, on day 5, and at the end of the study, on day 12, comparing these to the communities present in untreated mice. Community composition was assessed through 16S rRNA gene sequencing of fecal material. The microbiota of untreated mice analyzed on day 0 consisted mainly of bacteria from the order of Bacteroidales including the families of Bacteroidaceae*,* Muribaculaceae*,* Prevotellaceae and Rikenellaceae*.* Lachnospiraceae from the order of Eubacteriales was the main group of Gram-positive bacteria identified. The family of Enterobacteriaceae, which includes *E. coli*, represented less than 2% of total bacteria in all but one untreated mouse (Figure 6A). On day 5, after antibiotic treatment, microbial diversity was strongly decreased and mainly consisted of Enterobacteriaceae and Bacteroidaceae (Figure 6B). Of note, the microbial composition of mice gavaged with PBS or with recombinant *E. coli* EHV2 only had minor biological differences. At the end of the study, all mouse groups showed similar microbial compositions with dominance of Lachnospiraceae, Bacteroidaceae and Enterobacteriaceae (Figure 6C). The similarity of the microbial composition of the mouse groups before and after DSS treatment was illustrated by Bray Curtis-based principal coordinate analysis. Despite statistically significant differences between bacterial communities in the treatment groups, the clusters largely overlapped on day 5 before DSS treatment (Figure 6D) and after the development of colitis at the end of the study (Figure 6E).

**FIGURE 7:**
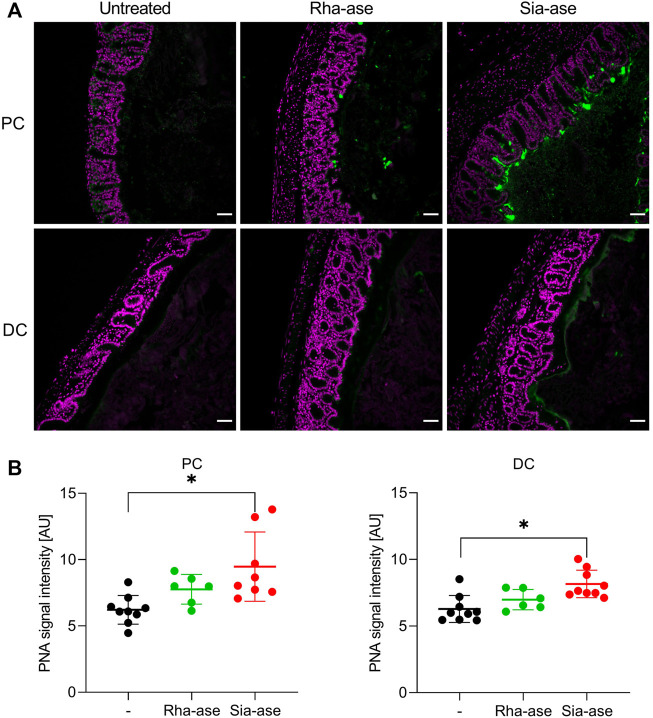
PNA lectin staining of proximal and distal colon tissue. **(A)** DAPI (violet) and PNA staining (green) in proximal colon (PC) and distal colon (DC) of untreated mice and mice with increased rhamnosidase and sialidase activity, scale bar 50 µm. **(B)** Quantification of PNA signal intensity was performed using ImageJ. Data are shown as mean ± s.d. (n = 6–9). *p ˂ 0.05 vs. untreated control by one-way ANOVA with Dunnett Post-test.

Given that supplementation of mice with recombinant *E. coli* expressing different GHs did not lead to biologically significant changes in the gut microbiota composition of the treatment groups, we deduced that the exacerbation of colitis observed in mice with increased sialidase activity was unlikely related to dysbiosis itself. Increased sialidase activity in the colon may also alter the occurrence of sialylated structures, thereby affecting cell functions relying on the recognition of sialic acid. To confirm the impact of increased sialidase activity on the distribution of sialylated glycans in the colon, we investigated the loss of sialic acid in mice supplemented with Rha-ase and Sia-ase expressing *E. coli* EHV2 by staining colon tissue with peanut agglutinin (PNA). PNA binds to the carbohydrate epitope Galβ1,3GalNAc (Chacko and Appukuttan, 2001; Corfield, 2018), which is often masked by terminal sialic acid. Mice with increased sialidase activity indeed presented elevated PNA reactivity in proximal and distal colon tissue in comparison to mice with increased rhamnosidase activity and mice that were gavaged with PBS instead of recombinant *E. coli* EHV2 (Figure 7A). The PNA signal was localized to the mucus layer, which coincides with the expected localization of Sia-ase released from recombinant *E. coli* EHV2. Quantitative analysis of the PNA signal in multiple sections of proximal and distal colon tissue confirmed the increase observed in mice supplemented with Sia-ase expressing *E. coli* EHV2 (Figure 7B).

**FIGURE 8:**
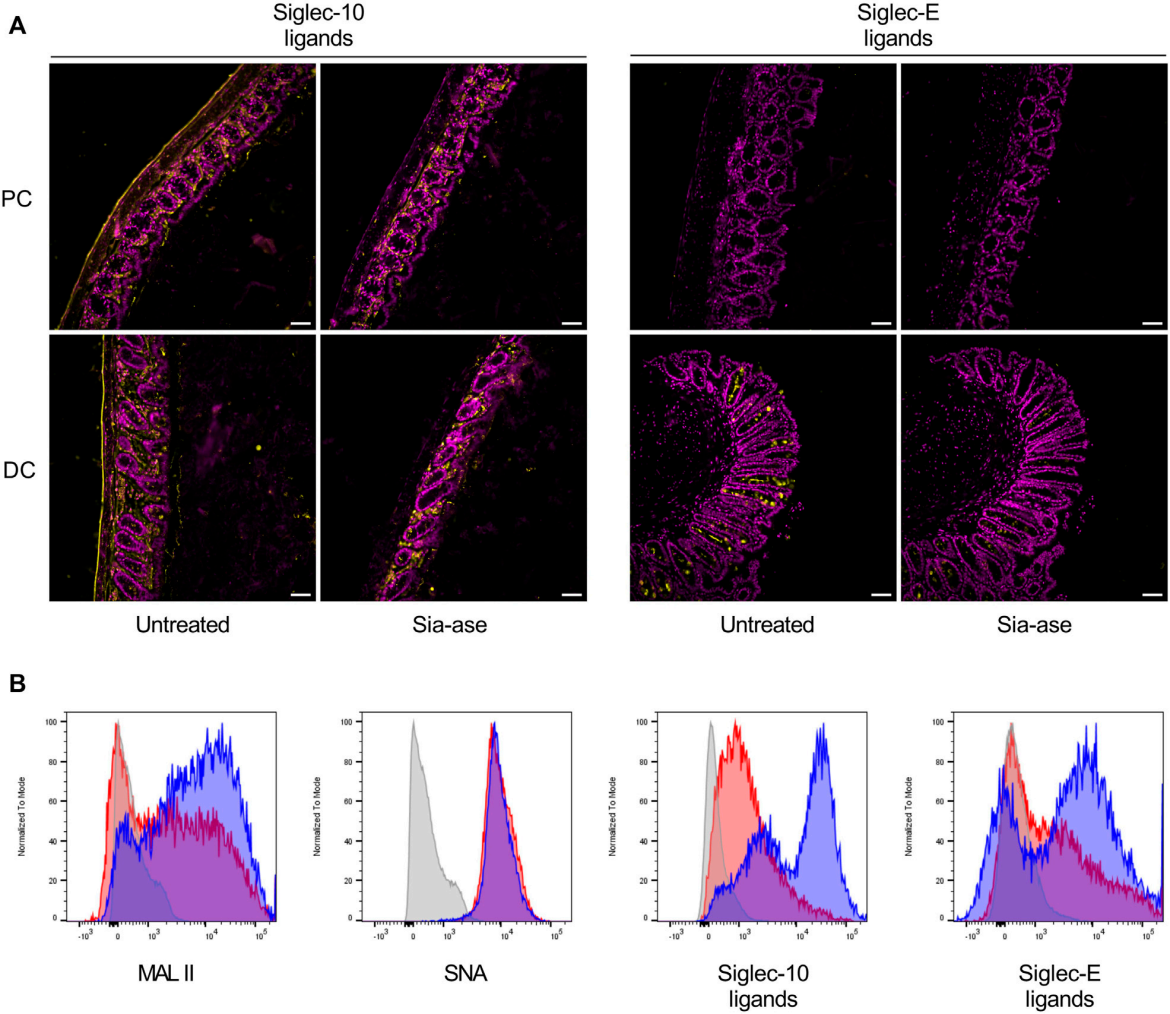
Effect of *in vitro* Sia-ase treatment on distribution of siglec ligands. **(A)** DAPI (violet) and Siglec-10 and siglec-E ligand (yellow) staining of proximal (PC) and distal colon (DC) sections from wild type mice treated with 200 µL of pure Sia-ase (34 μg ml^−1^) or PBS as a negative control over night at room temperature, scale bar 50 µm. **(B)** Flow cytometry analysis of lamina propria-derived CD11c^+^ dendritic cells. Cells treated with 50 µL of pure Sia-ase (34 μg ml^−1^) for 2 h at room temperature were stained for MAL II lectin binding, SNA lectin binding, and siglec-10 and -E binding. DCs incubated with secondary antibody alone (grey), DCs incubated with lectin or siglec (blue), DCs incubated with lectin or siglec after Sia-ase treatment (red).

In addition to the cleavage of sialic acid from mucosal glycans, increased sialidase activity may also decrease the presence of sialic acid on resident leukocytes in the colon mucosa, thereby possibly altering functions related to proteins interacting with surface sialic acid. Siglecs are signaling proteins mainly expressed on leukocytes, which down-regulate activation of immune cells after binding to their sialylated ligands (Läubli and Varki, 2020). The sialylated ligands to several siglecs are broadly exposed in the colon mucosa and submucosal connective tissue. For example, ligands to siglec-10 are found in the lamina propria and muscular wall of the proximal and distal colon, while ligands to siglec-E are mainly found in goblet cells in the distal colon (Figure 8A). Incubation of colon tissue sections with Sia-ase resulted in the disappearance of these siglec ligands, indicating that this sialidase was able to cleave sialic acid critically needed for siglec activation. Given the upregulation of pro-inflammatory properties of DCs due to reduction of siglec ligands (Chen et al., 2011; Pillai et al., 2012; Lubbers et al., 2018), we investigated the ability of Sia-ase to decrease surface sialylation of lamina propria-derived DCs. Incubation of isolated CD45^+^/CD11c^+^/MHCII^+^ DCs with Sia-ase decreased the binding of MAL II lectin, but not of SNA binding (Figure 8B), showing that Sia-ase was able to cleave α2,3-linked sialic acid, but not α2,6-sialic acid. Sia-ase treatment of DCs also resulted in strongly decreased siglec-10 and siglec-E binding (Figure 8B). Therefore, the exacerbation of induced colitis in Sia-ase supplemented mice is likely related to the remodeling of surface sialylation on immune cells found in the colon mucosa.

## Discussion

Our previous work showed that increased sialidase activity in the mouse colon enables the expansion of Enterobacteriaceae, thereby exacerbating inflammation in acute colitis (Huang et al., 2015). In the present study, the pro-inflammatory effect of sialidase activity has also been expanded to the remodeling of host mucosal glycans, which results in decreased presentation of siglec ligands. The oral supplementation of recombinant *E. coli* EHV2 overexpressing GHs enabled to assess the contribution of specific rhamnosidase, fucosidase and sialidase activity on the course of inflammation *in vivo*. The resulting increase in the respective GH activities in the colon of supplemented mice clearly showed the validity of the approach to delineate the impact of a given enzyme on the development of colitis. The colonization and intestinal expansion of recombinant *E. coli* EHV2 itself did not affect the severity of the disease as shown by the parallel course of colitis observed between untreated and *E. coli*-supplemented mice with increased rhamnosidase activity.

The absence of pathogenic effect of *E. coli* EHV2 supplementation itself on colitis also indicated that the sole expansion of *E. coli* in the gut of treated mice is not sufficient to intensify intestinal inflammation as suggested in our previous study (Huang et al., 2015). An obvious limitation of bacterial identification based on targeted sequencing of regions of the 16S rRNA gene is the lack of resolution beyond the genus or species level (Johnson et al., 2019). Strain-specific features such as virulence factors and bacteriocin expression, which may be critical for disease development, are overlooked when only considering changes in the microbiota composition at the level of 16S rRNA gene sequences. Along this line, carbohydrates released from the intestinal mucosa may also regulate the expression of virulence factors in addition to mediating the expansion of specific bacterial taxa. For example, increased free fucose levels in the colon has been shown to repress the expression of virulence genes in enterohemorrhagic *E. coli*, thereby promoting its colonization of the intestinal mucosa (Pacheco et al., 2012). Similarly, sialic acid released by sialidase activity may exacerbate colon inflammation through the regulation of virulence factors beyond the expansion of *E. coli* itself.

In addition to releasing sialic acid from the intestinal mucosa, bacterial sialidase activity can interfere with the surface glycosylation of host cells, such as resident immune cells. Sialic acid on host glycans is often protected from cleavage by enteric bacterial sialidase through O-acetylation (Corfield et al., 1993). Sialic acid O-acetylation is especially widespread in the gastrointestinal tract (Visser et al., 2021). The *B. vulgatus* sialidase applied in our study was anyway capable of removing sialic acid from the intestinal mucosa, as shown by increased PNA binding, and from lamina propria DCs. The loss of MAL II binding detected on sialidase-treated DCs confirmed the preference of the *B. vulgatus* sialidase for α2,3-linked sialic acid, which is prominently represented among siglec ligands (Gonzalez-Gil and Schnaar, 2021). The decrease in siglec ligand presentation may therefore contribute to lowering the immune-damping activity of siglecs in the context of DSS-induced colitis, thus contributing to the increased inflammatory response observed in mice with increased sialidase activity. The significance of sialidase activity on the abundance of siglec ligands and thus the immunoregulatory potential of siglecs has already been shown in the context of polybacterial sepsis (Chen et al., 2011) and *Streptococcus pneumonia* infections (Chang et al., 2012). Of note, mammalian sialidases have also been associated with regulation of intestinal inflammation. The host-encoded sialidase NEU3 has been shown to trigger colitis through the desialylation of intestinal alkaline phosphatase, which contributes to the detoxification of bacterial lipopolysaccharide (Yang et al., 2017; Yang et al., 2021). The induction of endogenous *Neu3* expression may therefore also contribute additively to the overall sialidase activity observed in mice supplemented with *E. coli* EHV2 expressing Sia-ase.

Intestinal inflammation has been shown to increase fucosylation in the small intestine, which leads to increased release of fucose into the intestinal lumen (Pickard et al., 2014). This increased availability of fucose decreases the expression of virulence genes and attenuates colitis induced by *Citrobacter rodentium* infection (Pham et al., 2014), possibly through shifts in bacterial composition. The increase of mucosal fucosylation results from the up-regulation of the α1,2-fucosyltransferase 2 *Fut2* gene in intestinal epithelial cells (Goto et al., 2014; Pickard et al., 2014). Interestingly, the *R. gnavus* fucosidase ATCC_03833 applied in our study mainly cleaves α1,2-linked fucose, the product of the FUT2 enzyme, yet we did not observe any effect of increased fucosidase activity on the severity of colitis in treated mice*.* This absence of any effect observed in mice with increased fucosidase activity may be related to the unchanged levels of fucosylation in the colon, where inflammation is localized in contrast to the reported up-regulation of *Fut2* in the small intestine (Goto et al., 2014; Pickard et al., 2014). Also, as observed in mice with increased sialidase activity, increased fucosidase activity did not change the bacterial composition between the treatment groups, suggesting that the severity of colitis was not affected by the microbial composition itself in our mouse model. Whereas fucosidase activity possibly influences the integrity of fucosylated glycans on host cells, the removal of fucose is not expected to alter siglec binding, although additional work is needed to confirm this interpretation. Furthermore, other glycan modifications, such as sulfation that is prominently found in the intestinal mucus (Benjdia et al., 2011), should also be investigated to better understand interactions between the host mucus and mucin-degrading bacteria. Accordingly, beyond the GH activities investigated in our study, the supplementation of mice with recombinant *E. coli* EHV2 expressing carbohydrate-active enzymes can be broadly applied to assess the functional significance of further activities including glycosulfatases, sialate O-acetylesterases and hexosaminidases in the remodeling of colonic glycosylation and glycan ligands to carbohydrate-binding signaling proteins.

## Materials and methods

### Mouse model of acute colitis

All animal experiments conducted in this study were performed in compliance with the Swiss Animal Protection Ordinance and approved by the Veterinary Office of the Canton of Zurich, Switzerland. C57BL/6 8- to 10-week-old male mice were treated with neomycin (1 g L^−1^), vancomycin (0.5 g L^−1^), ampicillin (1 g L^−1^) plus aspartame (0.2%, w/v, Sigma) in drinking water for 2 days. During this period, mice were gavaged daily with 150 µL of antibiotic solution containing neomycin (1 g L^−1^), vancomycin (0.5 g L^−1^), ampicillin (1 g L^−1^) and metronidazole (1 g L^−1^) plus aspartame (0.2%, w/v, Sigma). After 1 day of recovery, mice were gavaged on two consecutive days with 250 µL (5·10^8^ CFU ml^−1^) of recombinant *E*. *coli* EHV2 suspension in PBS. Mice were then treated with 3.5% (w/v) of DSS (molecular weight 36–50 kDa, MP Biomedicals) in drinking water for 5 days, followed by a supply of normal water until euthanasia by CO_2_ inhalation on day 12 or when body weight reached the threshold of 85% of the starting weight. All supplementations and DSS treatments were performed in three independent experiments.

### Glycoside hydrolase assays

Substrate specificity of purified GHs was assayed using the colorimetric substrates 4-nitrophenyl (NP)-α-d-xylopyranoside (Carbosynth), 4-NP-β-d-xylopyranoside (Sigma), 4-NP-α-l-arabinopyranoside (Carbosynth), 4-NP-β-l-arabinopyranoside (Carbosynth), 4-NP-α-l-rhamnopyranoside (Sigma), 4-NP-α-l-fucopyranoside (Carbosynth), 4-NP-β-l-fucopyranoside (Carbosynth), 4-NP-α-d-mannopyranoside (Carbosynth), 4-NP-β-d-mannopyranoside (Carbosynth), 4-NP-α-d-glucopyranoside (Sigma), 4-NP-β-d-glucopyranoside (Sigma), 4-NP-α-d-galactopyranoside (Sigma), 4-NP-β-d-galactopyranoside (Carbosynth), 4-NP N-acetyl-α-d-glucosamine (Carbosynth), 4-NP N-acetyl-β-d-glucosamine (Sigma), 4-NP N-acetyl-α-d-galactosamine (Sigma) and 4-NP N-acetyl-β-d-galactosamine (Sigma). Cell lysates of recombinant *E. coli* and mouse caecum fluid were centrifuged at 16,000 x g for 20 min at 4°C and supernatants were used for GH activity assays. Assays with 4-NP-substrates consisted of 10 µL of supernatant, 50 µL of 7 mM 4-NP-carbohydrate substrate and 190 µL of 100 mM sodium-phosphate buffer, pH 6.5. Samples were incubated at 37°C for 30 min and assays were stopped by adding ice-cold 250 µL of 500 mM Na_2_CO_3_, pH 10.5. Cleaved 4-NP was detected at 405 nm using a microplate reader (Tecan Infinite M200 Pro). For sialidase activity assay, 10 µL of supernatant was incubated with 190 µL of 0.1 mM fluorogenic substrate 2’-(4-methylumbelliferyl)-α-D-N-acetylneuraminic acid (4MU-NeuNAc, Carbosynth) in 100 mM Tris-Cl buffer, pH 7.4 for 15 min at 37°C. Assay was stopped by addition of ice-cold 800 µL of 500 mM Na_2_CO_3_, pH 10.5 and further diluted 40-fold prior to fluorescence measurement in a microplate reader (Tecan Infinite M200 Pro) at an excitation wavelength of 360 nm and an emission wavelength of 440 nm.

### Transepithelial permeability assay

Mice fasted for 4 h were gavaged with 600 mg kg^−1^ body weight of FITC-dextran (MW 3000-5000, Sigma). After 1 h, whole blood was collected by cardiac puncture under anesthesia using ketamine 65 mg kg^−1^ (Vetoquinol AG), xylazine 13 mg kg^−1^ (Graeub AG), acepromazine 2 mg kg^−1^ (Arovet AG) and allowed to clot for 30 min at room temperature (RT). Blood serum was collected after centrifugation at 1,500 *g* for 20 min at 4 °C. Serum fluorescence was measured using a multi-detection microplate reader (Tecan Infinite M200 Pro) with excitation wavelength of 485 nm and an emission wavelength of 535 nm. Autofluoresence of blood serum was subtracted by measuring the fluorescence of untreated blood serum.

### Bacterial DNA isolation and quantitative PCR

DNA was isolated from fecal pellets using the QIAamp DNA stool mini kit (Qiagen) according to manufacturer’s instructions for pathogen detection. Copy number of total bacterial 16S rRNA was determined by qPCR using the oligonucleotides 5′-GTG CCA GCM GCC GCG GTA A-3’ (515f) and 5′-GAC TAC CAG GGT ATC TAA T-3’ (805r) (Wasimuddin et al., 2020) mixed with KAPA SYBR Fast master mix (Sigma) and amplified with a CFX95 Touch™ Real-Time system (BioRad). Conditions were 50 cycles at 95°C for 30°s and 56°C for 50 s after an initial denaturation at 95°C for 3 min. The total reaction volume of 23 µL contained 2 µL of spermine (Sigma) dissolved in ddH_2_O at a concentration of 0.16 g L^−1^ to overcome polymerase inhibition by DSS (Krych et al., 2018).

### Molecular cloning and purification of glycoside hydrolases

The BVU_4143 sialidase from *Bacteroides vulgatus* (UniProt A6L7T1) was subcloned from the pET16b expression vector (Novagen) generated previously (Huang et al., 2015) into the constitutive expression vector pSF_OXB20 (Sigma) and transformed into *E. coli* EHV2 (Huang et al., 2015). The BT_1001 rhamnosidase from *Bacteroides thetaiotaomicron* (UniProt Q8A916) was cloned by PCR from *B. thetaiotaomicron* genomic DNA using the oligonucleotides BT_1001 for 5′-ATA CTC GAG ATG ATA CTT TTG GGT GCC TTG TCA TTG G -3′ and BT_1001 rev 5′-ATA GGA TCC CTA CAA ACG ATA GGT AAC AAT CCT TTC-3′, which introduced *XhoI* and *BamHI* used for cloning into pET16b. PCR conditions were 34 cycles of 10 s at 98°C, 20 s at 64°C, 1 min at 72°C and 5 min at 72°C final elongation after an initial denaturation at 98°C for 30 s. The fucosidase ATCC_03833 from *Ruminococcus gnavus* (UniProt A7B8B7) was also cloned by PCR from genomic DNA using the oligonucleotides RGN_16710 for 5′-ATA CTC GAG AAT CAG GAA ATA TGG AAT CGG ACC-3′ and RGN_16710 rev 5′-CAG GGA TCC TCG GAT GTA AAG TCC TAT TTT TTC-3′ including *XhoI* and *BamHI* sites for cloning into pET16b. PCR conditions were 30 cycles of 10 s at 98°C, 20 s at 64°C, 40 s at 72°C and 5 min at 72°C final elongation after an initial denaturation at 98°C for 30 s. The pET16b based constructs were transformed into *E. coli* BL21 (DE3) cells (Invitrogen) cultured in LB broth supplemented with ampicillin (100 μg/ml) at 37°C. Upon reaching an OD_600_ of 0.6 protein expression was induced by adding isopropyl β-d-1-thiogalactopyranoside to a final concentration of 1 mM and incubating bacteria at 37°C for 4 h. Bacteria were harvested by centrifugation at 3,000 *g* for 20 min. Resulting cell pellet was resuspended in 50 mM Tris-HCl, 100 mM NaCl plus 20 mM imidazole and lyzed by sonication (Digital Sonifier, Branson). Lysates were incubated with Ni Sepharose 6 Fast Flow (GE Healthcare Bio-Sciences AB, Uppsala) overnight at 4°C, washed with 50 mM Tris-HCl, 100 mM NaCl plus 40 mM imidazole and eluted with 50 mM Tris-HCl, 100 mM NaCl plus 500 mM imidazole. Proteins were dialyzed using Pur-A-Lyzer™ Maxi Dialysis kit (MW cut off: 6–8 kDa, Sigma) against a buffer containing 50 mM Na_2_HPO_4_, 100 mM NaCl and 0.005% Tween20 overnight at 4°C. All GHs were sublcloned from pET16b into pSF_OXB20 using *NcoI* (ThermoScientific) and *BamHI* (ThermoScientific). The constitutive expression vectors pSF_OXB20 containing the three GHs were transformed into *E. coli* EHV2 for oral gavage in mice.

### Inflammatory marker mRNA quantitative PCR

Mouse colon tissue was homogenized using a Precellys^®^ 24 tissue homogenizer (Bertin Instruments) and RNA was isolated using Trizol reagent (Sigma) according to manufacturer’s instructions. Total RNA (1 µg) was reverse transcribed using High-Capacity cDNA Reverse transcription Kit (Applied BioSystems™) according to manufacturer’s instructions. For quantitative PCR reactions KAPA SYBR^®^ Fast (Sigma) master mix was used and amplified with a CFX95 Touch™ Real-Time System with each 100 nM of the following primers: iNOS for: 5′-AGC CTT GCA TCC TCA TTG GG-3’; iNOS rev: 5′-CCT TTG AGC CCT TTG TGC TG-3’; TNFα for: 5′-GGC CTC CCT CTC ATC AGT TC-3’; TNFα rev: 5′-CAC TTG GTG GTT TGC TAC GAC-3’; IL-1β for: 5′-GCT GGA GAG TGT GGA TCC CAA G-3’; IL-1β rev: 5′-TGC TGA TGT ACC AGT TGG GG-3’; IL-6 for: 5′-CAC GGC CTT CCC TAC TTC AC-3’; IL-6 rev: 5′-GCC ATT GCA CAA CTC TTT TCT C-3’; TGFβ for: 5′-TGG AGC AAC ATG TGG AAC TC-3’: TGFβ rev: 5′-GTC AGC CGG TTA CCA-3’; Occludin for: 5′-CCC TGA CCA CTA TGA AAC AG-3’; Occludin rev: 5′-TTG ATC TGA AGT GAT AGG TG-3’. After an initial denaturation at 95°C for 3 min, cycling conditions were 50 cycles at 95°C for 10 s and 60°C for 30 s. The ∆∆c_t_ method was applied to analyze the relative gene expression with β-actin as a normalization reference and samples from untreated mice were used as control samples. To overcome polymerase inhibition by DSS, 5 µL spermine (Sigma) dissolved in ddH_2_O at a concentration of 0.16 g L^−1^ was added to the 20 µL total reaction volume in the reverse transcription and qPCR reactions (Krych et al., 2018).

### Histological scoring of colon tissue

Mouse colon tissue was fixed in 10% neutral buffered formalin (Sigma) for 24 h and embedded in paraffin. Tissue samples were cut into 5 µm thick sections, deparaffinized and stained with hematoxylin and eosin (Sigma). Histological alterations were scored individually by an independent investigator blinded to the sections’ origin as follows: normal epithelium morphology (0), isolated loss of goblet cells (1), loss of goblet cells in large areas (2), loss of isolated crypts (3), loss of crypts in large areas (4). Leukocyte infiltration was also graded as no infiltrate (0), infiltrate around crypt basis (1), infiltrate reaching to *lamina muscularis mucosae* (2), extensive infiltration reaching the *lamina muscularis mucosae* and thickening of the mucosa with abundant oedema (3), infiltration of the *lamina submucosa* (4). The total score represents the sum of the epithelium and infiltration score.

### Histochemistry

Isolated colon tissue was tightly rolled up on itself (Swiss roll) in a histological cassette and incubated for 2 h at RT in methacarn (60% methanol, 30% chloroform and 10% acetic acid) fixation solution to preserve the mucus layer (Fernandez et al., 2019). Tissue was subsequently washed for 1 h in methanol, dehydrated and paraffinized. After deparaffinization and rehydration, sections of 5 µm thickness were blocked with 0.5% gelatin (Sigma) in Carbo-free™ blocking solution (Vector Laboratories) for 1 h at RT, followed by staining with 15 μg ml^−1^ of fluorescein-labelled PNA (Vector Laboratories) and 0.1 μg ml^−1^ of DAPI (Sigma) in solution containing 1x Carbo-free™ blocking solution and 1x lectin staining buffer consisting of 110 mg L^−1^ CaCl_2_ (Sigma), 200 mg L^−1^ MgCl_2_ (Sigma) and 200 mg L^−1^ MnCl_2_ x 4 H_2_O (Sigma) for 1 h at RT in the dark. For staining with siglecs, tissue sections were subjected to antigen retrieval by cooking for 20 min in citrate buffer pH 6.0. Tissue sections were incubated over night with 200 µL of pure Sia-ase (34 μg ml^−1^) or PBS as a negative control at RT. After blocking in Carbo-free™ blocking solution for 1 h at RT, 5 μg ml^−1^ of siglec-10 (RD Systems) or 5 μg ml^−1^ of siglec-E (RD Systems) were coupled to fluorescently labelled secondary antibody, goat F (ab’)2 anti-human IgG-Fc Dylight^®^ 550 (Abcam) or PE anti-mouse IgG2a antibody (BioLegend), respectively, for 1 h at RT in the dark with a dilution of 1:100. Tissue sections were then stained with the siglec-secondary antibody complexes for 1 h at RT in the dark. Fluorescence was acquired using a Leica DMi8 microscope with ×20 objective. Quantification of PNA signal was performed using ImageJ (Schneider et al., 2012). PNA signal was determined by selecting areas of the same size in the mucus layer. The mean PNA intensity was then calculated using a threshold of four to exclude the background.

### Isolation of lamina propria leukocytes

Isolated colon tissue was washed three times with HBSS (Gibco) containing 2% FBS, then incubated in 1 mM DTT (Thermo Scientific) in HBSS 2% FBS at 37°C under constant agitation for 15 min. Tissue was then incubated in HBSS 2% FBS 5 mM EDTA (Sigma) for 15 min at 37°C under constant agitation. This process was repeated twice with fresh buffer. Tissue was cut into smaller pieces and incubated with RPMI (Gibco), containing 2% FBS and 3 mg ml^−1^ collagenase type IV (Sigma) and incubated for 10 min at 37°C under constant agitation. Enzymatic digestion was stopped by adding ice cold RPMI 2% FBS. Cell suspension was first filtered through a 100 μm cell strainer (Greiner) followed by a 40 μm cell strainer (Falcon). Cells were centrifuged at 350 x g for 10 min at 4°C and resuspended in RPMI 2% FBS at a density of 1.5 10^6^ cells ml^−1^.

### Flow cytometry

Cells isolated from the lamina propria (1.5 10^6^ cells ml^−1^) were treated with 50 µL of pure Sia-ase (34 μg ml^−1^) for 2 h at RT in RPMI 2% FBS. The sialidase reaction was stopped by adding three times the amount of ice cold RPMI 2% FBS. Cells were centrifuged at 350 x g for 10 min at 4 °C and resuspended in FACS buffer (PBS, 2% FBS, 5 mM EDTA) or lectin staining buffer (PBS, 1% FBS, 0.1 mM CaCl2). Cells were first blocked with TruStain FcX™ PLUS anti-mouse CD16/32 (BioLegend) (1:100) for 15 min on ice. Siglec-E at 5 μg ml^−1^ (R&D Systems) and siglec-10 at 5 μg ml^−1^ (R&D Systems) were coupled to secondary antibodies PE anti-mouse IgG2a (BioLegend) or PE anti-human IgG Fc (BioLegend), respectively, with a dilution of 1:100 for 30 min at RT in the dark. Biotinylated lectin MAL II at 5 μg ml^−1^ (Vector Laboratories) was first coupled to FITC streptavidin 1:100 (BioLegend) for 30 min at RT. Cells were incubated on ice for 40 min with siglec-Fc probes, followed by 20 min incubation with the lectins and the remaining staining panel consisting of violet-fluorescent reactive dye (Invitrogen, 1:1,000), CD11c (BioLegend, 1:100), CD45 (BioLegend, 1:200), MHC II (BioLegend, 1:100) and the lectin SNA at 5 μg ml^−1^ (Vector Laboratories). After incubation, cells were washed with FACS buffer or lectin staining buffer, centrifuged at 350 x g for 10 min at 4°C and resuspended in 200 µL FACS buffer or lectin staining buffer. Samples were analyzed with BD FACSCanto™ II Flow Cytometer (BD Biosciences).

### Next-generation sequencing

Bacterial DNA was isolated from fecal content with QIAamp Fast DNA Stool Mini Kit (Qiagen) and amplified using modified primers targeting the V4-V5 region of the 16S ribosomal gene (515F; 5′-GTGYCAGCMGCCGCGGTAA-3′, 926R; 5′-CCGYCAATTYMTTTRAGTTT-3′). For amplicon PCR total volume was 25 μL, containing 7 µL of molecular grade water, 2 µL of spermine (Sigma) at 0.16 g L^−1^, 12.5 µL of KAPA HiFI HotStart ReadyMix (Roche), 1.25 µL of each forward and reverse primer at a concentration of 10 µM and approximately 10 ng bacterial DNA. Cycling conditions were 3 min at 95°C followed by 25 cycles of 20 s at 98°C, 30 s at 56°C, 25 s at 72°C, then by a final extension for 1 min at 72°C. Resulting PCR products were purified using SPRI magnetic beads (AMPure XP, Beckman Coulter) according to the manufacturer’s protocol with a ratio of 0.8-fold volume of AMPure XP beads per volume of PCR product. Next, the purified amplicon DNA was dual-indexed using the Nextera XT Index kit set A. For indexing PCR total volume was 25 μL, containing 7.5 µL of purified PCR product, 12.5 µL KAPA HiFI HotStart ReadyMix (Roche), 2.5 µL of each index 1 and index 2. Cycling conditions were as follows: one step for 3 min at 95°C followed by 12 cycles of 20 s at 98 °C, 30 s at 55°C. 25 s at 72°C then 1 min at 72°C. The indexed PCR products were immediately purified with AMPure XP beads with a ratio of 0.8-fold volume of AMPure XP beads per volume of PCR product. The libraries were then normalized to 2 nM, pooled, denatured with 0.2 N NaOH, diluted with HT1 hybridization buffer (Illimuna, Inc.) to a final concentration of 8 p.m. A PhX sequencing control (Illumina, Inc.) was incorporated for a total concentration of 12.5 p.m. PhiX in the 8 p.m. library. Sequencing was carried out on an Illumina MiSeq FGx sequencing platform (Verogen, Inc.) with the MiSeq Reagent kit V3 (Illumina, Inc.), generating paired-end reads of 2 × 300 bp. The raw reads were processed using Cutadapt (v3.7) (Martin, 2011) to remove primer sequences, followed by quality control and denoising with DADA2 (1.18) (Callahan et al., 2016). The parameters for DADA2 were the following; truncLen: Forward read: 260, reverse read: 240; maxN: 0; maxEE: 2.2; truncQ: 2 and rm. phix: TRUE. Taxonomy assignment was carried out using DECIPHER against the SILVA SSU r138 (2019) database until the genus level. The abundance tables for the amplicon sequence variants (ASVs) and taxonomy assignments were imported into phyloseq (McMurdie and Holmes, 2013) for downstream rarefaction, alpha and beta diversity analyses.

## Data Availability

The datasets presented in this study can be found in online repositories. The names of the repository/repositories and accession number(s) can be found below: https://www.ncbi.nlm.nih.gov/sra/PRJNA901913
