# Supplementary material for: Increase of intestinal bacterial sialidase activity exacerbates acute colitis in mice
Source: Front Mol Biosci. 2022 Dec 9;9:1075459. doi: 10.3389/fmolb.2022.1075459 (PMC9780602; doi:10.3389/fmolb.2022.1075459)
Supplement: Supplementary file 1 [file DataSheet1.PDF]

Supplementary Material

Supplementary Figures

Activity assays revealed that these GHs are also active on their annotated targets when expressed constitutively in *E. coli*. The enzymes are not secreted, but their activity is still observable in the supernatant (not shown) due to bacterial lysis. Among the three GHs the Rha-ase showed the lowest activity in the cell lysate with about 18 pmol min^{-1} μl^{-1}, while the Fuc-ase showed the highest activity with about 40 pmol min^{-1} μl^{-1}, the activity of the Sia-ase was about 30 pmol min^{-1} μl^{-1}. The control, consisting of *E. coli* EHV2 transformed with an empty plasmid, showed no activity.

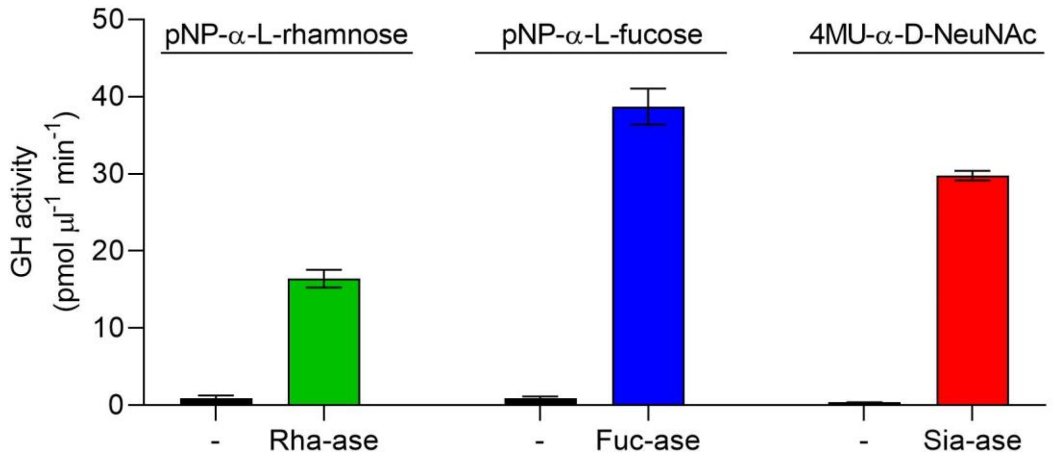

Figure S1. Glycoside hydrolase activity in cell lysate of recombinant *E. coli* EHV2 constitutively expressing target Rha-ase, Fuc-ase and Sia-ase. The substrates applied in the assays are marked at the top. *E. coli* EHV2 transformed with an empty plasmid was used as negative control (-). n=3. Data are shown as mean ± s.d.

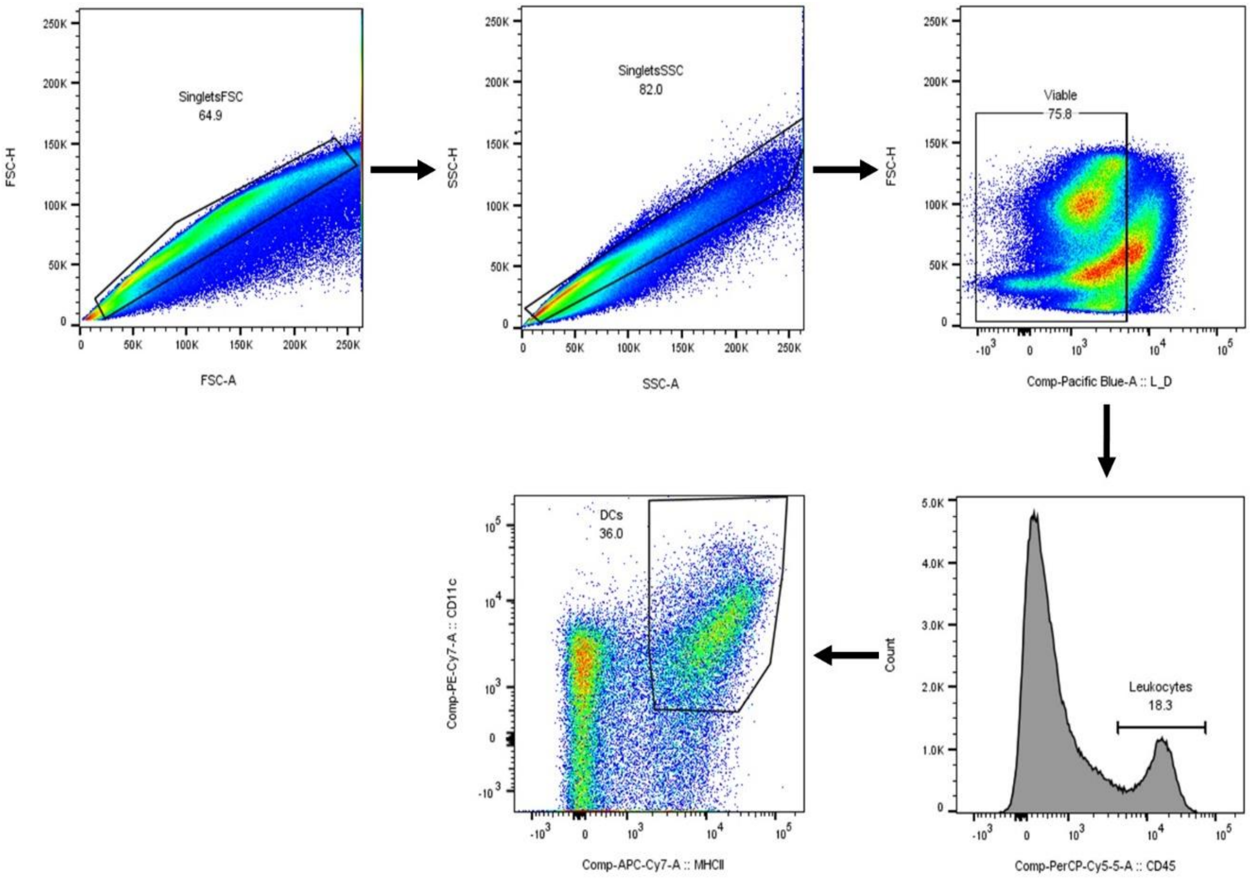

Figure S2. Gating strategy for flow cytometry analysis of DCs. DCs were gated the following way: First singlet cells were selected by FSC, then singlets by SSC, followed by low intensity of live/dead staining. Viable leukocytes were gated as CD45 positive and DCs were subsequently defined as MHCII and CD11c positive cells
